# Economic and environmental assessment of bacterial poly(3-hydroxybutyrate) production from the organic fraction of municipal solid waste

**DOI:** 10.1186/s40643-021-00392-4

**Published:** 2021-05-19

**Authors:** Jon Kepa Izaguirre, Leire Barañano, Sonia Castañón, José A. L. Santos, M. Teresa Cesário, M. Manuela R. da Fonseca, Itziar Alkorta, Carlos Garbisu

**Affiliations:** 1 NEIKER-Basque Institute of Agricultural Research and Development, Basque Research and Technology Alliance (BRTA), Parque Científico y Tecnológico de Bizkaia, P812, 48160 Derio, Spain; 2 iBB – Institute for Bioengineering and Biosciences, Instituto Superior Técnico, Av. Rovisco Pais, 1049-001 Lisboa, Portugal; 3 Department of Biochemistry and Molecular Biology, University of the Basque Country (UPV/EHU), P.O. Box 644, 48080 Bilbao, Spain

**Keywords:** Bioeconomy, Bioplastics, *Burkholderia sacchari*, Circular economy, Polyhydroxyalkanoate production

## Abstract

The management of municipal solid waste is a major logistic and environmental problem worldwide. Nonetheless, the organic fraction of municipal solid waste (OFMSW) is a valuable source of nutrients which can be used for a variety of purposes, according to the Circular Economy paradigm. Among the possible applications, the bioproduction of a biodegradable polyester, poly(3-hydroxybutyrate) [P(3HB)], using OFMSW as carbon platform is a promising strategy. Here, an economic and environmental assessment of bacterial P(3HB) production from OFMSW is presented based on previously published results. The SuperPro Designer® software was used to simulate P(3HB) production under our experimental parameters. Two scenarios were proposed depending on the fermentation medium: (1) enzymatic hydrolysate of OFMSW supplemented with glucose and plum waste juice; and (2) basal medium supplemented with glucose and plum waste juice. According to our results, both scenarios are not economically feasible under our experimental parameters. In Scenario 1, the low fermentation yield, the cost of the enzymes, the labour cost and the energy consumption are the factors that most contribute to that result. In Scenario 2, the cost of the extraction solvent and the low fermentation yield are the most limiting factors. The possibility of using process waste as raw material for the generation of other products must be investigated to enhance economic feasibility. From an environmental viewpoint, the photochemical oxidation potential (derived from the use of anisole as extraction solvent) and the generation of acid rain and global warming effect (caused by the burning of fuels for power generation) are the most relevant impacts associated to P(3HB) production under our experimental parameters.

**Supplementary Information:**

The online version contains supplementary material available at 10.1186/s40643-021-00392-4.

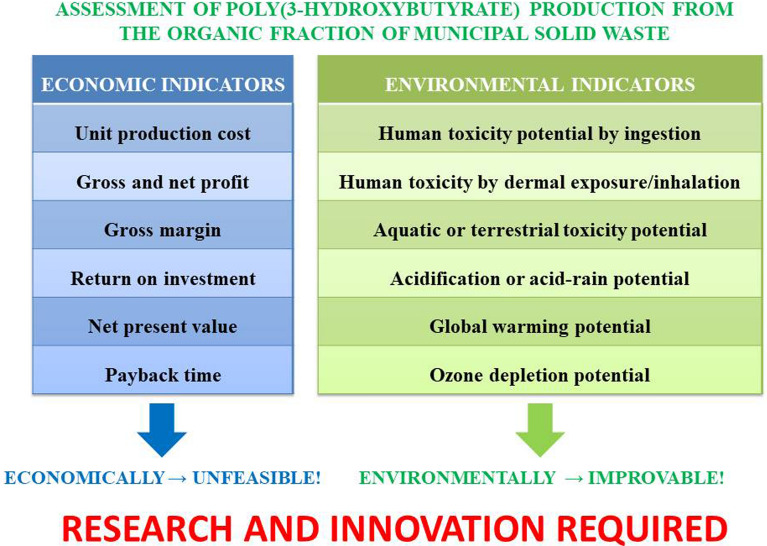

## Introduction

Due to the development of science and technology, many industrial and commercial activities have undergone great growth and innovation in the last decades. Regrettably, such growth has been accompanied in many cases by an adverse environmental impact and, in particular, the generation of extremely large volumes of wastes. In many cases, such wastes have been safely handled, managed, treated, disposed of at an appropriate waste facility, reused, etc., but in many more cases they have been simply, even illegally, dumped in the environment with well-known negative consequences for the recipient ecosystems and human health. An example of this is the generation of very large amounts of municipal solid waste (MSW), which has grown massively in recent decades, being currently a logistic and environmental problem of much concern worldwide. The *Circular Economy* paradigm aims at transforming our current, unsustainable linear economic model (i.e. the take–make–waste model), and hence our society, by (i) progressively decoupling economic activity from the consumption of finite resources and (ii) designing waste out of the system, according to three core principles: design out waste and pollution; keep products and materials in use; and regenerate natural systems (Ellen MacArthur Foundation). One of the ultimate goals of the Circular Economy approach is to minimize the generation of waste, maintaining the value of products, materials, and resources for as long as possible. Likewise, relatively recently, the concept of *Bioeconomy*, based on the utilization of renewable biological resources and waste streams to produce food, feed, materials and energy has been emphasized and supported as a way to achieve a more sustainable, greener economic model which can boost the creation of new value chains, while protecting biodiversity and the environment. The merging of these two “Guiding Principles” (Circular Economy and Bioeconomy) in the current economy arena has led to the term *Circular Bioeconomy* focused on a sustainable, resource-efficient valorization of biomass in integrated production chains and chain networks, while making use of wastes and optimizing (ideally, considering the three pillars of sustainability) the value of biomass over time via cascading, in an attempt to retain resource quality by adhering to the bio-based value pyramid and the waste hierarchy where feasible and suitable (Stegman et al. 2020).

Polyhydroxyalkanoates (PHAs) are aliphatic polyesters accumulated intracellularly by many prokaryotic organisms as carbon and energy storage in response to nutrient imbalance (Olaya-Abril et al. 2017). PHAs form a large family of biodegradable bioplastics (Kabir et al. 2017) which can be tailored to offer properties similar to those of several plastics manufactured from petroleum sources (Możejko-Ciesielska and Kiewisz 2016). In fact, polyhydroxyalkanoates have often been highlighted as competitors of petroleum-derived plastics due to their physical properties, biocompatibility and biodegradability, which makes them very attractive for the development of biomaterials such as, for instance, supports for protein immobilization (Bello-Gil et al. 2018), a procedure long used for improving the performance of enzymatic reactions in many industrial applications (Alkorta et al. 1996, 1998). Moreover, PHAs are the only bioplastics that are degradable in the marine environment (Rujnić-Sokele and Pilipović 2017; DiGregorio 2009). This explains why the focus on PHAs has greatly increased in the last decades. The most common PHA is poly(3-hydroxybutyrate) [P(3HB)], which typically depicts melting point and tensile strength values similar to those of polypropylene (Abe and Doi 2001). The production of P(3HB) from the organic fraction of municipal solid waste (OFMSW) appears to be a promising strategy for the valorization of OFMSW according to the Circular Bioeconomy principles (Izaguirre et al. 2019) and, in particular, fits the goal of obtaining bio-derived, biodegradable plastics from especially abundant wastes.

Municipal solid waste typically includes residential, commercial, institutional, municipal, industrial and construction wastes, which are habitually collected by the municipality. The world production of MSW is estimated around 2,000 million tons per year, with 34–53% of that amount corresponding to the organic fraction, mainly made up of food scraps, paper, garden, forest trimmings and alike (Abad et al. 2019; Braguglia et al. 2018; Qin et al. 2017). Landfill disposal, incineration, composting and anaerobic digestion for biogas production are some of the most widely used treatments for OFMSW. However, despite recent technological improvements in waste management and treatment, the sad reality is that the generation of MSW (and, hence, OFMSW) continues to increase worldwide, with concomitant adverse environmental impacts such as the emission of greenhouse gases, the discharge of potentially toxic leachates to the environment, the emergence and dissemination of animal diseases, etc. (Demichelis et al. 2017).

Interestingly, due to its nutrient-rich composition, abundance and low cost, OFMSW has great potential as feedstock for the production of biochemicals, biofuels and other biomaterials (Battista et al. 2020). Barampouti et al. (2019) reviewed the production of bio-ethanol, bio-diesel and biogas from OFMSW. Ghanavati et al. (2015) and Izaguirre et al. (2019) studied the use of OFMSW to produce lipids and P(3HB), respectively. Compared to other organic wastes, OFMSW presents the advantage of being produced daily in all cities, i.e. high and constant availability throughout the year. Another advantage is that, since OFMSW is generated in the cities themselves, its transportation to processing sites, which are usually located in peri-urban industrial areas, has a lower cost and a reduced environmental impact (Shahzad et al. 2013).

The design of suitable strategies for an efficient utilization of OFMSW in biotechnological and industrial production processes is critical for its successful implementation since, among other aspects, they can significantly influence the economic cost of the process. In this respect, the use of mild process conditions and procedures, such as enzymatic hydrolysis, for the release of the nutrients present in the biomass (e.g. OFMSW) can prevent the formation of undesirable inhibitors, thereby increasing process yield. Unfortunately, this can sometimes result in conflicting outcomes: for instance, the use of enzymes (for the enzymatic hydrolysis of the biomass) can be highly expensive (Amit et al. 2018), creating a conflict between whether it is better to use enzymatic hydrolysis and thus produce a larger quantity of product, or whether one should opt for stronger process conditions and, hence, avoid the economic costs associated to the purchase of enzymes. Apart from these direct economic costs, other aspects must be taken into consideration, e.g. the assignment of OFMSW to the production of biodegradable plastics will not only reduce the amount of MSW to be managed and the well-known environmental problem of plastic pollution, but will also help to alleviate our excessive dependence on petroleum-based products.

One of the relevant expenses of many industrial fermentation processes is the cost of the organic substrate required for the microbial cultivation to take place. The possibility of using organic wastes, such as OFMSW, as fermentation substrate can result in large cost savings, thereby improving the economic feasibility of the process (de Paula et al. 2017; Obruca et al. 2015). In any event, the economic feasibility of a given process depends on many factors, e.g. substrate and material costs, labour costs, plant location, environmental regulations, taxes applied, etc. To this purpose, economic analyses serve to assess the feasibility of a given process. In many cases, these economic studies are based on data obtained previously at lab-scale and pilot-scale, which are then analysed by a simulation software, such as, for instance, SuperPro Designer® and Aspen Plus®.

In this work, an economic and environmental assessment of the feasibility of producing P3HB from OFMSW was performed. To this purpose, *Burkholderia sacchari* DSM 17165 (a bacterial strain capable of simultaneously using glucose, xylose and arabinose as carbon source) was selected for the fermentation step, due to its proven potential for P(3HB) production (Cesário et al., 2014; Izaguirre et al., 2019; Raposo et al., 2017). This evaluation, based on lab-scale data previously published (Izaguirre et al. 2019), was performed with SuperPro Designer® software, considering all the steps of the P(3HB) production process.

## Materials and methods

### Process simulation

In this study, a process for bacterial P(3HB) production from OFMSW was economically and environmentally evaluated. Mass and energy requirements for the P(3HB) production process were estimated by performing a simulation with the aid of the commercial software SuperPro Designer 10®. For this simulation, the following conditions and specifications were used: (1) the P(3HB) production plant would be built in The Basque Country (Spain); (2) its lifetime would be 20 years; (3) the construction *plus* start-up phase would take one year; (4) the waste processing capacity of the P(3HB) production plant would be 1 ton day^−1^; and (5) the plant would operate for 330 days year^−1^ (the rest of the time, up to 365 days, the plant would be stopped for maintenance and cleaning works).

According to the Spanish Ministry for the Ecological Transition (MITECO 2017), each year 877 thousand tons of OFMSW are generated in Spain, of which 20% (175.4 thousand tons) are produced in The Basque Country. In Spain, OFMSW is selectively collected and, subsequently, 70% is derived to produce compost and biogas, while the remaining 30% is disposed of in landfills or incinerated.

### Simulation scenarios

The industrial plant simulated in this study is based on a bacterial P(3HB) production process using *Burkholderia sacchari* DSM 17165, carried out in the fed-batch mode, which was developed and reported in previous works (Izaguirre et al. 2019, 2020). Here, two scenarios, based on the fermentation medium, were considered to assess the economic and environmental feasibility of the bacterial P(3HB) production process: (1) in Scenario 1 (Additional file 1: Fig. S1A), those sugars present in OFMSW were initially released by the combination of a thermo-chemical pre-treatment and an enzymatic hydrolysis. Subsequently, the enzymatic hydrolysate was used as culture medium for the fermentative production of P(3HB). During the cultivations, glucose (a lower amount compared to Scenario 2) and sugar-rich plum waste juice were added as feed to enhance productivity; (2) in Scenario 2 (Additional file 2: Fig. S2B), the enzymatic hydrolysate from OFMSW was not used as fermentation medium. Instead, fermentation was initiated with a basal medium which contains salts and glucose (for a detailed description of its composition, see Izaguirre et al. 2019). Plum waste juice was added as feed solution after the batch period. Finally, in each scenario, the P(3HB) produced was extracted. By comparing both scenarios (Scenarios 1 and 2 with and without OFMSW hydrolysate, respectively), one can evaluate to what extent the use of OFMSW, as source of nutrients for the fermentation process, contributed to the performance of the P(3HB) production process represented in Additional file 1: Fig. S1. In this Additional file 1: Fig. S1, for simplification purposes, pieces such as valves and piping are omitted from the flowsheet. Nonetheless, they were taken into account for the economic assessment.

### Description of the P3HB production process

The P(3HB) production process simulated here included three main steps: (i) thermo-chemical pre-treatment and enzymatic hydrolysis of OFMSW; (ii) fermentation; and (iii) extraction–separation (Izaguirre et al. 2019, 2020).

The OFMSW, kindly provided by a local composting company (EPELE, Gipuzkoa, Spain) was composed of pre-screened domestic and garden wastes (Izaguirre et al. 2019). After removing impurities (i.e. stones, plastics, glass, etc.), OFMSV was ground, using a coffee grinder (Moulinex AR100), and subsequently mixed with a 1% H_2_SO_4_ solution at a solid-to-liquid ratio of 13.5% (w/v). Afterwards, the waste was pre-treated at 121 °C for 60 min in a blending tank (V-101). This thermo-chemical pre-treatment is intended to break up the lignocellulosic structure of the biomass, so that it becomes more accessible to enzymatic hydrolysis. The resulting mixture was cooled and then neutralized with NaOH, following Izaguirre et al. (2019). After neutralization, the mixture was enzymatically hydrolyzed in a reactor (R-101) at 50 °C for 24 h using a blend of 90 mg g^−1^ Pentopan 500 BG and 150 mg g^−1^ Celluclast BG. Non-hydrolyzed solids were separated by centrifugation in a disk-tank centrifuge (DS-101), and the supernatant (hydrolysate) was used for the next step, i.e. the bacterial cultivation. As mentioned above, the thermo-chemical pre-treatment and the enzymatic hydrolysis described here were only contemplated in Scenario 1.

For the bacterial production of P(3HB), the enzymatic hydrolysate was heat-sterilized and transferred to a fermenter (FR-101), which was inoculated with *Burkholderia sacchari* DSM 17165 (10% v/v) previously grown for 12 h in Luria–Bertani (LB) broth in a 2000-mL Erlenmeyer stirred in an orbital shaker at 170 rpm, 30 ºC (Izaguirre et al. 2019). The fermentation process was carried out aerobically at pH = 6.8 and 32 °C in a stirred tank bioreactor for 50 h. The solid fraction was separated from the liquid fraction by centrifugation in a disk-tank centrifuge (DS-102). Finally, the P(3HB)-enriched bacterial biomass was lyophilized in a freeze dryer (FDR-101).

For the extraction–separation step, we followed the procedure reported by Rosengart et al. (2015). Briefly, the lyophilized biomass was transferred to a blending tank (V-102), which contained anisole at 120 °C, and mixed for 30 min. The extraction solvent (anisole) alters cell permeability and then dissolves the released P(3HB). After extraction, the remaining biomass was separated by filtration in a rotary vacuum filter (RVF-101). Finally, the separation of P(3HB) from the extraction solvent (anisole) was carried out by crystallization in a continuous crystallizer (CR-101). For the economic and environment assessment presented here, it was assumed that the evaporated solvent was collected to then be re-used in subsequent extractions.

### Economic analysis

The economic evaluation of both scenarios was performed using SuperPro Designer 10® software, through which the total capital cost, annual production cost and revenue generation can be estimated.

The total capital cost of the P(3HB) production plant is dependent on three different parameters: direct fixed capital, working capital, and start-up validation cost. The direct fixed capital includes equipment purchase costs, as well as other direct and indirect costs related to the construction of the plant, such as piping, insulation and engineering, among others. The contribution (%) of each component was estimated, based on total equipment purchase cost, using several multipliers (Petrides 2015). For the economic analysis, the price of the equipment was gathered from reputable websites. In this study, and since the local government (Basque Government) strongly promotes and enables the creation of new industries and facilities, the cost of the land was not taken into account in the economic evaluation. The contribution of the working capital and start-up validation cost to the total capital cost was 1.5 and 5% of the direct fixed capital, respectively.

Concerning annual production costs, raw materials (namely, enzymes, solvents, salts, carbon sources, etc.) usually have a most important contribution to these costs, but they also include other costs derived from maintenance and repair, labour, utilities, quality control, consumables, waste disposal and so on. For the economic assessment, the cost of raw materials and consumables was obtained from reputable suppliers of laboratory equipment, reagents, etc. In our specific case, OFMSW (and its transportation to the P3HB production plant) was kindly provided by a local composting company (EPELE, Gipuzkoa, Spain). Likewise, the plum waste juice was freely provided by CATAR-CRITT Agro Ressources (France) and, thus, its cost was not included in the economic evaluation. The so-called “facility-dependent costs” correspond to depreciation of the fixed capital investment, equipment maintenance costs, insurance, taxes and other general expenses. Our P(3HB) production plant was designed to operate for 20 years and, according to this, the straight-line method was used to calculate capital depreciation. Equipment maintenance and repair cost were estimated to be 1% of the direct fixed capital. In accordance with the local legislation, insurance and taxes were estimated to be 0.04 and 1.38% of the direct fixed capital, respectively. Labour costs basically consist of the salaries of operators and engineers, to which corresponding taxes must be added. For the correct operation of the envisioned plant, in Scenario 1, six operators and two engineers were considered necessary, while four operators and two engineers were estimated for Scenario 2. The estimated salary of an operator and an engineer was US$ 26,000 and US$ 41,000 year^−1^, respectively. The cost of the quality control was estimated to be 15% of total labour cost. The cost of waste disposal and treatment of gaseous emissions was estimated to be 0.013 and 0.002 US$ kg^−1^, respectively. Finally, the level of consumption of materials and energy was estimated according to the mass and energy balance calculated by the simulation software (unit costs were obtained from supplier companies).

Under both scenarios, the main revenue comes from the sale of the produced P(3HB). The market for green, bio-derived, biodegradable bioplastics, such as P(3HB) and PLA, is very promising, specially taking into consideration the increasing awareness of the negative impact of petroleum-derived, non-biodegradable plastics on the environment (Dhaman and Ugwu 2013). Poly(3)hydroxybutyrate is probably the most studied polyhydroxyalkanoate due to a variety of promising characteristics (Dhaman and Ugwu 2013): (i) its material properties are comparable to those of polypropylene; (ii) it can be synthesized from renewable low-cost feedstocks; (iii) its synthesis can be operated under mild process conditions with minimal environmental impact; (iv) many different microbial strains are known to produce P3HB; (v) it can be degraded aerobically and anaerobically without forming toxic products; and (vi) it can be used as biomaterial for medical applications and packaging, among other uses. At present, the market price of P3HB is 4000 US$ ton^−1^ (Ramos et al. 2019; Stavroula et al. 2020).

In addition, in Scenario 1, after the enzymatic hydrolysis, the undigested OFMSW was sold as biofertilizer at a price of 0.01 US$ kg^−1^. Also, a waste management remuneration (0.077 US$ kg^−1^) was received from the Basque Government, as it is currently promoting and encouraging Circular Economy initiatives.

The economic feasibility of the bacterial P(3HB) production process was evaluated according to several indicators calculated by SuperPro Designer 10® software: gross and net profit, gross margin, return on investment, net present value, and payback time. Gross profit is the revenue from which the annual operating cost has been subtracted, while net profit also considers the depreciation, income tax (20% in The Basque Country), and similar costs. The return on investment (ROI) evaluates the viability of the investment, according to the following equation:

$$ {\text{ROI }}\left( \% \right) = \frac{{\text{Net profit Total investment}}}\times 100. $$

The net present value (NPV) measures the profitability of the production process in absolute net terms (thus, it allows one to know whether the investment will bring profits or not). A positive NPV value means that, a priori, the planned investment should make a profit. The NPV can be calculated according to the following equation (Van Dael et al. 2015):

$$ {\text{NPV}} = \mathop \sum \limits_{n - 1}^{T} \frac{{{\text{CF}}_{n} }}{{\left( {1 + i} \right)^{n} }} - I_{0} , $$

where *T* = lifetime of the investment; CF_*n*_ = difference between revenues and costs in year *n*; *I*_*0*_ = initial investment; and *i* = discount rate.

The payback time, or time required to recover the capital investment, is calculated as follows:

$$ {\text{Payback time}} \left( {{\text{years}}} \right) = \frac{{\text{Total investment}}}{{\text{Net profit per year}}}. $$

Finally, in order to determine cash flow patterns during the lifetime of the P3HB production plant, cumulative cash flows were calculated. The patterns were plotted using Microsoft Excel 2010. Similarly, various discount rates (see below) were considered to assess their possible effect on profitability.

### Environmental assessment

In order to estimate the potential environmental impact (PEI) of the bacterial P3HB production process, an algorithm developed by the U.S. Environmental Protection Agency was used (i.e. WAR tool). This algorithm, based on the calculation of PEI, is divided into eight impact categories: human toxicity potential by ingestion (HTPI); human toxicity potential by dermal exposure and inhalation (HTPE); aquatic toxicity potential (ATP); acidification or acid rain potential (AP); terrestrial toxicity potential (TTP); photochemical oxidation potential (PCOP); global warming potential (GWP); and ozone depletion potential (ODP).

## Results and discussion

### Economic assessment

The estimated total capital cost for the bacterial P3HB production was US$ 7,418,949 and US$ 5,549,004 for Scenario 1 and 2, respectively (see below). As abovementioned, this figure encompasses the direct fixed capital, the working capital cost and the start-up validation cost. The direct fixed capital included the purchase of equipment and its installation, the engineering cost, the cost of building the P(3HB) production plant, and other related costs. The size and number of the different components and equipment were estimated based on the mass and energy balance of the simulated P(3HB) production process. Table 1 details the bare minimum equipment used in Scenario 1 and 2, as well as corresponding characteristics and costs. Total equipment cost was US$ 1,066,000 and US$ 764,000 for Scenario 1 and 2, respectively. This difference is due to the fact that, in Scenario 2, the thermo-chemical pre-treatment and the enzymatic hydrolysis steps were not performed, as this scenario did not include the use of OFMSW hydrolysate as fermentation medium. Under both scenarios, the equipment that contributed most to the total cost was the fermenters (FR-101), in agreement with Kwan et al. (2015), Leong et al. (2017) and Mudliar et al. (2008). In fact, the bacterial cultivation was the time-limiting step of the process as a whole and, therefore, to cover a 24 h production period, it was necessary to instal three fermenters.

**Table 1:** Purchase cost of the main equipment for Scenario 1 and 2

Code	Name	Units	Capacity/	Cost (US$)	
			Size	Scenario 1	Scenario 2
GR-101	Grinder	1	3,000 kg h^−1^	26,000	0
V-101	Blending tank	1	10,000 L	26,000	26,000
R-101	Stirred reactor	3	10,000 L	135,000	0
FR-101	Fermenter	3	15,000 L	300,000	300,000
ST-101	Heat sterilizer	1	1,681 L h^−1^	20,000	20,000
ST-102	Heat sterilizer	3	52 L h^−1^	60,000	0
SFR-101	Shake-flask rack	12	2 L	10,000	10,000
SR-101	Seed reactor	1	1,500 L	35,000	35,000
FDR-101	Freeze dryer	1	433 kg	41,000	41,000
V-102	Blending tank	1	15,000 L	54,000	54,000
RVF-101	Rotary vacuum filter	1	15 m^2^	52,000	52,000
CR-101	Crystallizer	1	5,000 L	30,000	30,000
AF-101	Air filter	1	32,000 L h^−1^	1,000	1,000
AF-102	Air filter	3	460,000 L h^−1^	3,000	3,000
DS-101	Disk-stack centrifuge	1	2,174 L h^−1^	30,000	0
DS-102	Disk-stack centrifuge	1	1,839 L h^−1^	30,000	30,000
DE-101	Dead-end filter	1	46 m^2^	0	10,000
	Unlisted equipment			213,000	152,000
Total				1,066,000	764,000

Table 2 shows other direct and indirect costs related with the building of the bacterial P(3HB) production plant. The working capital, i.e. those expenses derived from the initialization of the P(3HB) production plant and the operational training, was estimated at 1.5% of the direct fixed capital. Specifically, the start-up cost was estimated to be US$ 106,293 and US$ 61,843 for Scenario 1 and 2, respectively.

**Table 2:** Costs related with the building of the P(3HB) production plant

Costs	Component	% DFC*	Cost (US$)	
			Scenario 1	Scenario 2
Direct	Equipment purchase	15	1,067,000	759,000
	Installation	3	230,000	245,000
	Process piping	10	709,000	489,000
	Instrumentation	6	424,000	324,000
	Insulation	1	67,000	44,000
	Electrical facilities	2	137,000	101,000
	Buildings	6	388,000	307,000
	Yard improvement	2	137,000	103,000
	Auxiliary facilities	9	644,000	479,000
Indirect	Engineering	14	951,000	713,000
	Construction	19	1,331,000	998,000
Other	Contractor´s fee	5	316,000	228,000
	Contingency	8	565,000	368,000
		Total	6,966,000	5,158,000

*DFC* direct fixed capital

^*^The contribution (%) of each component was estimated, based on total equipment purchase cost, using several multipliers (Petrides 2015)

Table 3 shows the annual production and maintenance costs for both scenarios. The operating cost for Scenario 1 was 28% higher than for Scenario 2, in part due to the presence of the thermo-chemical pre-treatment and enzymatic hydrolysis steps in the former. The following factors contributed most to the higher production cost found for Scenario 1: the price of the enzymes, the heating/cooling utilities for the thermo-chemical pre-treatment, and the labour cost. In any case, under both scenarios, one of the main operating costs (approximately, 50% of the cost) was the abovementioned “facility-dependent costs”, which included expenses related to the use of the facility, namely, equipment maintenance, capital depreciation and other costs (insurance, taxes, etc.). Some utilities, such as the heat-transfer agents and the amount of power (energy) used, also contributed significantly to the operating cost, particularly under Scenario 1 (20%) as the thermo-chemical pre-treatment was performed at high temperature and involved several steps. The carbon source (i.e. substrate for bacterial fermentation) is normally a major cost of fermentation processes (Esteban and Ladero 2018; Rodríguez-Pérez et al. 2018). However, in this study, the cost of the plum waste juice was not taken into account, since it was kindly provided by CATAR-CRIT Agro Ressources (France). This plum waste juice (i.e. plum concentrate) is obtained by extrusion from fruit waste, making it an inexpensive and easily obtainable carbon source. The amount of waste produced was estimated by the software as part of the simulation. As indicated above, the cost of waste disposal and treatment of gaseous emissions was estimated to be 0.013 and 0.002 US$ kg^−1^, respectively.

**Table 3:** Annual production cost (US$) of the P(3HB) production plant for Scenario 1 and 2

	Scenario 1			Scenario 2		
	Quantity	US$/unit	US$/year	Quantity	US$/unit	US$/year
Raw materials						
OFMSW	357.0 t	− 77	− 27,489			
Ammonia	264.5 t	20	5290	264.5 t	20	5,290
Anisole	48.8 t	2120	103,456	59.1 t	2120	125,294
Celluclast	16.1 t	6270	100,947			
Glucose	65.6 t	400	26,240	137.0 t	400	54,800
NaOH for disinfection	9,539,3 t	10	95,393	9,000.1 t	10	90,001
Pentopan BG	9.3 t	9200	85,560			
Salts	19.6 t	800	13,280	23.6 t	800	18,880
NaOH	37.9 t	300	11,370			
Sulphuric acid	46.3 t	70	3241			
Sub-total			444,777			294,265
Consumables						
2000-mL shake flask	214 items	1.8	385.2	214 items	1.8	385.2
Filtration membrane				8 m^2^	400	3319
Sub-total			385.2			3704.2
Quality control						
Sub-total			19,605.0			26,995.0
Utilities						
Electricity	1,159,122 kW h^−1^	0.11	127,503.4	789,424.0 kW h^−1^	0.11	86,836.6
Steam	8738.0 t	12.00	104,856.0	4885.0 t	12.00	58,620.0
Cooling water	121,580.0 t	0.05	6079.0	75,747.0 t	0.05	3787.4
Glycol	509,864.0 t	0.35	178,452.4	132,002.0 t	0.35	46,200.7
Sub-total			411,490.8			195,444.7
Facility dependent						
Sub-total			969,449			761,943.0
Labour dependent						
Sub-total			245,065			194,598.0
Waste treatment						
Waste disposal	3682.3 t	13	47,869.9	3,745.4 t	13	48,690.2
Gaseous emissions	9749.6 t	2	19,499.2	10,051,7 t	2	20,102.0
Sub-total			67,369.1			68,792.2
Total cost			2,158,141.1			1,545,742.1

*t* tons

Table 3 shows the annual P(3HB) production, as well as the total annual revenue, for Scenarios 1 and 2. The annual P(3HB) production was 45 tons for Scenario 1 and 55 tons for Scenario 2. The total revenues obtained throughout the year in Scenario 1 and 2 were US$ 214,549 and US$ 220,000, respectively. In both scenarios, the main revenue came from the sale of P(3HB) (Table 4). In Scenario 1, there was an extra income from the sale of the residual biomass from the enzymatic hydrolysis as biofertilizer. In Scenario 2, a higher yield of P(3HB) was obtained, mainly due to the use of a basal medium (supplemented with glucose and plum waste juice) as fermentation medium. The unit price of the P(3HB) and the biofertilizer was 4 US$ kg^−1^ and 0.01 US$ kg^−1^, respectively. The waste management remuneration received from The Basque Government for the treatment of the OFMSW was 0.077 US$ kg^−1^. The unit production cost of P(3HB) was 48 US $ kg^−1^ P(3HB) for Scenario 1 and 28 US $ kg^−1^ P(3HB) for Scenario 2. The specific consumption of OFMSW was 8 kg kg^−1^ P(3HB). This specific consumption value was determined with the data obtained in Scenario 1, since the OFMSW was used as a sugar platform for the cultivation of bacteria only in this Scenario 1.

**Table 4:** Total annual revenue generated under Scenario 1 and 2

	Scenario 1			Scenario 2		
	Quantity Tons	Price US$/unit	Revenue US$/year	Quantity	Price US$/unit	Revenue US$/year
P(3HB)	45	4000	180,000	55	4000	220,000
Biofertilizer	706	10	7,060			
Remuneration	357	77	27,489			
Total			214,549			220,000

The profitability of both scenarios was evaluated according to several indicators whose value is presented in Table 5. Most importantly, we concluded that both scenarios were not economically feasible, since negative NPV and ROI values were obtained. Another factor that supports the lack of economic viability of the bacterial P(3HB) production process presented here was the production cost of P(3HB) which, under both scenarios, was much higher than its actual market price. Our P(3HB) production cost was clearly not competitive, compared to that reported by other authors (Al-Battashi et al. 2019; Dietrich et al. 2017; Vandi et al. 2018).

**Table 5:** Indicators for process profitability for Scenario 1 and 2

Indicator	Scenario 1	Scenario 2
Unit production cost (US$ year^−1^)	48	25
Gross profit (US$ year^−1^)	− 1,943,000	− 1,289,000
Net profit (US$ year^−1^)	− 1,282,000	− 793,000
Gross margin (%)	− 890	− 588
Return on investment (%)	− 17	− 14
Net present value at 7% (US$)	− 18,213,000	− 12,163,000
Payback time (years)	N/A	N/A

**Fig. 1:**
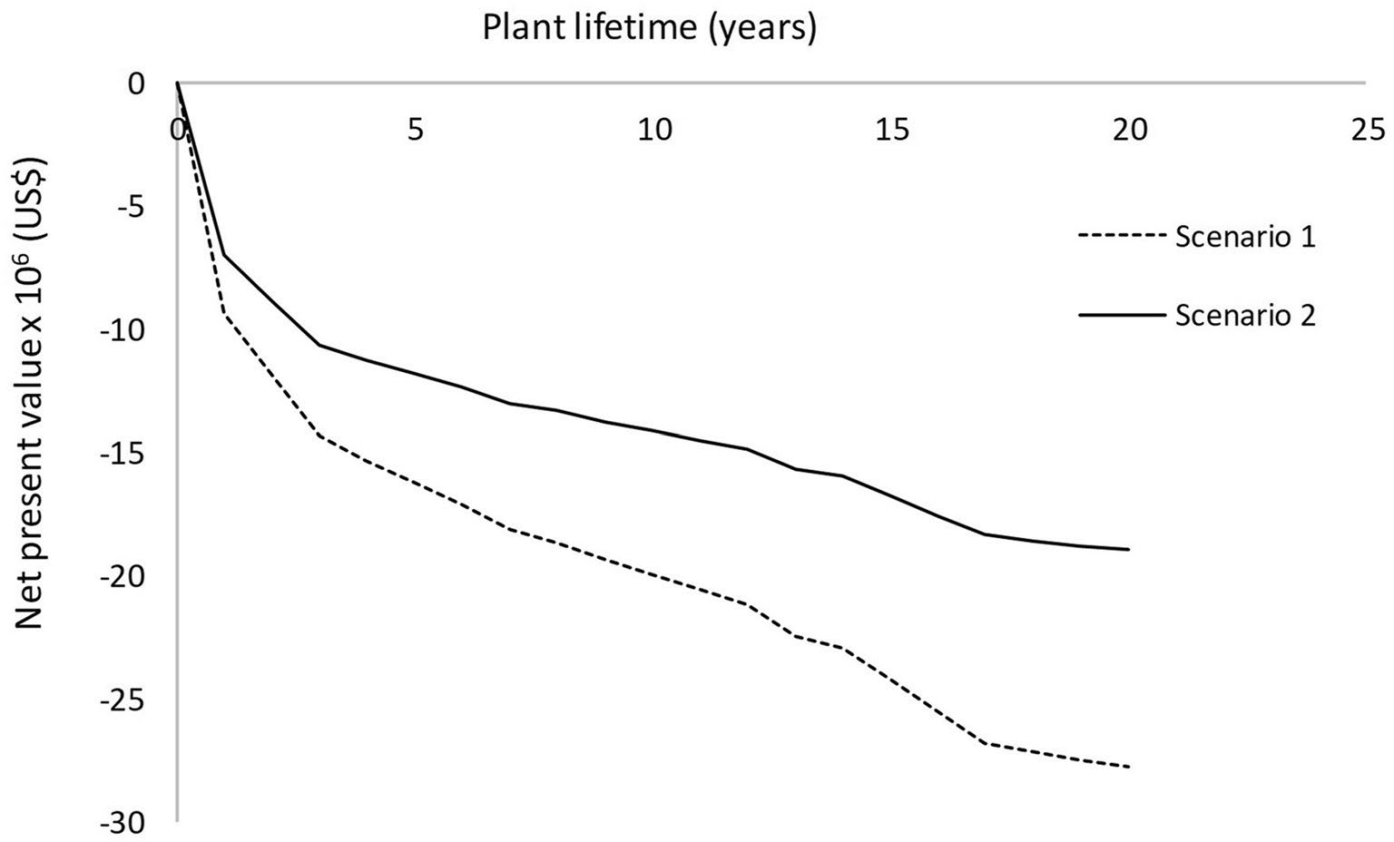
Net present value (NPV) of the bacterial P3HB production process throughout the plant´s lifetime for both scenarios

Under both scenarios, net present values remained negative throughout the plant´s lifetime (Fig. 1), indicating an unfavourable economic outlook in which expenses are higher than income. An important factor for the lack of economic viability of our bacterial P(3HB) production process was the use of OFMSW hydrolysate as fermentation medium. The hydrolysate production involved several steps (thermo-chemical pre-treatment, enzymatic hydrolysis) where expensive raw materials (enzymes) and high energy were required. The feasibility of process implementation by re-usable immobilized enzymes should be assessed. Another factor that weighed negatively under both scenarios was the low fermentation yield, which was, most likely, due to the fact that both OFMSW and the plum waste juice (plum concentrate) are very complex, heterogeneous substrates, made of a mixture of compounds. The use of complex substrates has been reported to adversely affect fermentation yields (Ghanavati et al. 2015; López-Gómez et al. 2019). Furthermore, the extraction–separation step also involved a considerable cost, especially owing to the large amounts of extraction solvent (anisole) required. In any case, in our simulation, we contemplated the recovery of the extraction solvent for its reuse in subsequent extractions. Solvent losses through evaporation were also taken into account for a more realistic scenario.

Our results clearly indicate that P(3HB) production by *Burkholderia sacchari* using complex waste streams as carbon source is not economically feasible due to the low productivities attained. This strain accumulates polymer preferentially under nutrient limitation (N or P) and excess of carbon. When using waste streams as carbon source which are also nutrient-rich, polymer production is thus compromised. In this case, to attain higher polymer productivities, the use of another strain that produces PHB in a growth-associated manner can be hypothesized. *Azohydromonas australica* (former *Alcaligenes latus*) grows fast and can accumulate PHB up to 80% of cell dry weight on sucrose (Gahlawat and Srivastava 2013). Besides, this strain produces P(3HB) based on various inexpensive substrates such as beet molasses, soya and malt wastes and maple sap. Although this strain can grow on glucose and fructose (Xie and Yokota 2005), its growth on xylose has not been reported. Therefore, to increase process profits, the use of the xylose-rich supernatant obtained after P(3HB) production (based on the glucose in the OFMSW hydrolysate) could be proposed, for example, for the production of furfural. Besides this liquid off-stream, the solid and gaseous emissions generated during the bacterial P(3HB) production process could be used for production of other valuable products such as biogas, CO_2_, H_2_, proteins, etc. Moreover, increasing the scale of the plant and the whole operation could also enhance profitability, as indicated by Choi (1999). Further, in this economic assessment, as it is usually the case, only those costs related with the production of P(3HB) have been taken into consideration. Nevertheless, it must also be considered that there are some co-benefits associated to the bacterial production of P3HB from OFMSW, e.g. (1) the use of a waste as raw material for a production process avoids the costs associated to its management, storage, disposal or treatment; (2) P(3HB) is a biodegradable material, which limits the costs associated to its management after useful life; and (3) the use of a biodegradable product, such as P(3HB), circumvents many of the well-known environmental costs associated to the use of non-degradable products (e.g. regular petroleum-based plastics). From a holistic point of view, all these and similar socioeconomic and environmental aspects should be taken into consideration, which would most likely modify the outcome of the assessment of the economic feasibility of the bacterial P(3HB) production process studied here.

### Environmental assessment

**Fig. 2:**
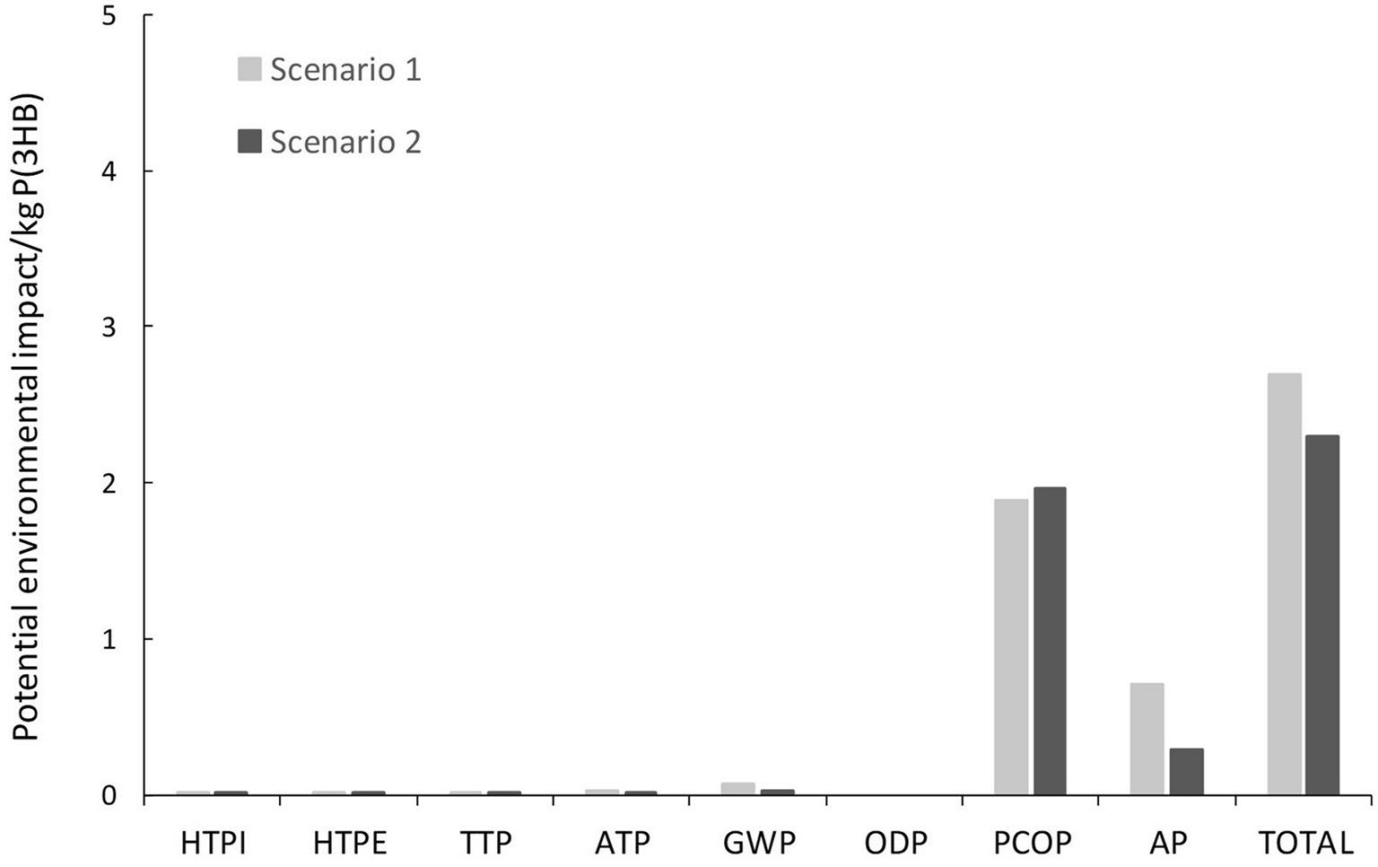
Potential environmental impact (PEI) per kg of P3HB produced. *HTPI* human toxicity potential by ingestion, *HTPE* human toxicity potential by dermal exposure and inhalation, *ATP* aquatic toxicity potential, *AP* acidification or acid rain potential, *TTP* terrestrial toxicity potential, *PCOP* photochemical oxidation potential, *GWP* global warming potential, *ODP* ozone depletion potential

As described above, the potential environmental impact of the bacterial P(3HB) production process was calculated using the WAR tool developed by the U.S. Environmental Protection Agency. The results were presented as “potential environmental impact leaving the system per kg of P(3HB) generated” (Fig. 2). The photochemical oxidation potential (PCOP), derived from the use of anisole as extraction solvent, generated the highest potential environmental impact. The burning of fuels for power generation (required for the plant's operation) also had a significant potential environmental impact, as reflected by the following two indicators: acid rain potential (AP) and global warming potential (GWP). Nevertheless, the potential environmental impact derived from the consumption of energy can be reduced by promoting the use of an energy mix with a higher percentage of renewable energy. During the bacterial P(3HB) production process, no relevant toxic products are used or generated and, therefore, the estimated potential toxicity for humans and/or aquatic and terrestrial ecosystems was very low. Halogenated hydrocarbons are not used either, so the potential impact on ozone depletion was zero. When comparing both scenarios, the potential environmental impact in terms of AP and GWP was higher in Scenario 1, presumably due to the higher energy consumption required for the thermo-chemical pre-treatment and enzymatic hydrolysis steps. By contrast, the potential environmental impact in terms of PCOP was higher in Scenario 2, owing to the higher anisole consumption.

## Conclusions

The OFMSW and fruit wastes (e.g. plum waste juice) are both residues generated in large quantities worldwide. Their sugar-rich composition makes them suitable raw materials for the production of high-value compounds. In addition, their re-entry into the value chain, according to the Circular Economy paradigm, is a step-forward towards sustainability. Based on this premise, in previous works, we designed and validated a process for the bacterial production of P(3HB) from these organic wastes. In the present work, an economic and environmental assessment of such P(3HB) production process was carried out to evaluate its feasibility. According to different economic feasibility indicators (gross and net profit, gross margin, return on investment, net present value), our bacterial P(3HB) production process was clearly unfeasible. Although the thermo-chemical pre-treatment and enzymatic hydrolysis steps performed under Scenario 1 had a great impact on economic costs, they were not the main cause of the process unfeasibility. Actually, the most relevant factor was the low fermentation yields, which were, at least partly, due to the high heterogeneity and complexity of the waste used as feedstock. Nonetheless, the use of the undigested OFMSW (from the enzymatic hydrolysis) and the residual biomass to produce other compounds would enhance the process profitability. Similarly, other aspects must be considered: (1) the use of wastes (OFMSW, plum concentrate) as raw materials for production processes avoids the costs associated to their management, storage, disposal or treatment; (2) P(3HB) is a biodegradable material, which limits the costs associated to its management after useful life; and (3) the use of a biodegradable product, such as P3HB, circumvents many of the well-known environmental costs associated to the use of non-degradable products (e.g. regular petroleum-based plastics). From a holistic point of view, all these and similar socioeconomic and environmental aspects should be taken into consideration, which would most likely modify the outcome of the assessment of the economic feasibility of the bacterial P(3HB) production process studied here. From an environmental point of view, the photochemical oxidation potential, derived from the use of anisole as extraction solvent, generated the highest potential environmental impact. The burning of fuels for power generation also had a significant potential environmental impact. Finally, we concluded that innovation is urgently required to enhance fermentation performance in order to increase the economic feasibility of bacterial P3HB production processes.

### Supplementary Information

**Additional Figure 1.** Flowsheet of the bacterial P3HB production process in Scenario 1. Green: thermo-chemical pre-treatment and enzymatic hydrolysis. Blue: bacterial process. Orange: extraction-separation.

**Additional Figure 2.** Flowsheet of the bacterial P3HB production process in Scenario 2. Green: thermo-chemical pre-treatment and enzymatic hydrolysis. Blue: bacterial process. Orange: extraction-separation.

## Data Availability

The simulation carried out in this study was based on data obtained previously at lab-scale (Izaguirre et al. 2019, 2020; Rosengart et al. 2015). All data generated or analysed during this study are included in this published article.

## References

[CR1] Abad V, Avila R, Vicent T, Font X (2019). Promoting circular economy in the surroundings of an organic fraction of municipal solid waste anaerobic digestion treatment plant: Biogas production impact and economic factors. Bioresour Technol.

[CR2] Abe H, Doi Y (2001) Bacterial polyesters. In: Buschow KHJ, Cahn RW, Flemings MC, IIschner B, Kramer EJ, Mahajan S (eds). Encyclopedia of Materials: Science and Technology, 2nd edn, pp 448–453. Elsevier

[CR3] Al-Battashi H, Annamalai N, Al-Kindi S, Nair AS, Al-Bahry S, Verma JP, Sivakumar N (2019). Production of bioplastic (poly-3-hydroxybutyrate) using waste paper as a feedstock: Optimization of enzymatic hydrolysis and fermentation employing *Burkholderia sacchari*. J Clean Prod.

[CR4] Alkorta I, Garbisu C, Llama MJ, Serra JL (1996). Immobilization of pectin lyase from *Penicillium italicum* by covalent binding to nylon. Enzyme Microbial Technol.

[CR5] Alkorta I, Garbisu C, Llama MJ, Serra JL (1998). Industrial applications of pectic enzymes: a review. Process Biochem.

[CR6] Amit K, Nakachew M, Yilkal B, Mukesh Y (2018). A review of factors affecting enzymatic hydrolysis of pretreated lignocellulosic biomass. Res J Chem Environ.

[CR7] Barampouti EM, Mai S, Malamis D, Moustakas K, Loizidou M (2019). Liquid biofuels from the organic fraction of municipal solid waste: a review. Renew Sustain Energy Rev.

[CR8] Battista F, Frison N, Pavan P, Cavinato C, Gottardo M, Fatone F, Eusebi AL, Majone M, Zeppilli M, Valentino F, Fino D, Bolzonella D, Bassham CB (2020). Food wastes and sewage sludge as feedstock for an urban biorefinery producing biofuels and added-value bioproducts. J Chem Technol Biotechnol.

[CR9] Bello-Gil D, Roig-Molina E, Fonseca J, Sarmiento-Ferrández MD, Ferrándiz M, Franco E, Mira E, Maestro B, Sanz JM (2018). An enzymatic system for decolorization of wastewater dyes using immobilized CueO laccase-like multicopper oxidase on poly-3-hydroxybutyrate. Microb Biotechnol.

[CR10] Braguglia CM, Gallipoli A, Gianico A, Pagliaccia P (2018). Anaerobic bioconversion of food waste into energy: a critical review. Bioresour Technol.

[CR11] Cesário MT, Raposo RS, de Almeida MCMD, van Keulen F, Ferreira BS, da Fonseca MMR (2014). Enhanced bioproduction of poly-3-hydroxybutyrate from wheat straw lignocellulosic hydrolysates. New Biotechnol.

[CR12] Choi MJ (1999). Factors affecting the economics of polyhydroxyalkanoate production by bacterial fermentation. Appl Microbiol Biotechnol.

[CR13] da Cruz Pradella JG, Taciro MK, Mateus AYP (2010). High-cell-density poly (3-hydroxybutyrate) production from sucrose using *Burkholderia sacchari* culture in airlift bioreactor. Bioresour Technol.

[CR14] de Paula FC, de Paula CBC, Gomez JGC, Steinbüchel A, Contiero J (2017). Poly(3-hydroxybutyrate-co-3-hydroxyvalerate) production from biodiesel by-product and propionic acid by mutant strains of *Pandoraea* sp. Biotechnol Prog.

[CR15] Demichelis F, Pleissner D, Fiore S, Mariano S, Navarro Gutiérrez IM, Schneider R, Venus J (2017). Investigation of food waste valorization through sequential lactic acid fermentative production and anaerobic digestion of fermentation residues. Bioresour Technol.

[CR16] Dietrich K, Dumont MJ, Del Rio LF, Orsat V (2017). Producing PHAs in the bioeconomy—towards a sustainable bioplastic. Sustain Prod Consum.

[CR17] DiGregorio BE (2009). Biobased performance bioplastic: Mirel. Chem Biol.

[CR18] Esteban J, Ladero M (2018). Invited review Food waste as a source of value-added chemicals and materials: a biorefinery perspective. Int J Food Sci Technol.

[CR19] Gahlawat G, Srivastava AK (2013). Development of a mathematical model for the growth associated Polyhydroxybutyrate fermentation by *Azohydromonas australica* and its use for the design of fed-batch cultivation strategies. Bioresour Technol.

[CR20] Ghanavati H, Nahvi I, Karimi K (2015). Organic fraction of municipal solid waste as a suitable feedstock for the production of lipid by oleaginous yeast *Cryptococcus aerius*. Waste Manag.

[CR21] Izaguirre JK, da Fonseca MMR, Fernandes P, Villarán MC, Castañón S, Cesário MT (2019). Upgrading the organic fraction of municipal solid waste to poly(3-hydroxybutyrate). Bioresour Technol.

[CR22] Izaguirre JK, da Fonseca MMR, Castañón S, Villarán MC, Cesário MT (2020). Giving credit to residual bioresources: from municipal solid waste hydrolysate and waste plum juice to poly (3-hydroxybutyrate). Waste Manag.

[CR23] Kabir MM (2017). Can bio-plastics replace non-biodegradable plastics. J Appl Biotechnol Bioeng.

[CR24] Kwan TH, Pleissner D, Lau KY, Venus J, Pommeret A, Lin CSK (2015). Techno-economic analysis of a food waste valorization process via microalgae cultivation and co-production of plasticizer, lactic acid and animal feed from algal biomass and food waste. Bioresour Technol.

[CR25] Leong YK, Show PL, Lan JCW, Loh HS, Lam HL, Ling TC (2017). Economic and environmental analysis of PHAs production process. Clean Technol Environ Policy.

[CR26] López-Gómez JP, Latorre-Sánchez M, Unger P, Schneider R, Coll Lozano C, Venus J (2019). Assessing the organic fraction of municipal solid wastes for the production of lactic acid. Biochem Eng J.

[CR27] Możejko-Ciesielska J, Kiewisz R (2016). Bacterial polyhydroxyalkanoates: still fabulous?. Microbiol Res.

[CR28] Mudliar SN, Vaidya AN, Suresh Kumar M, Dahikar S, Chakrabarti T (2008). Techno-economic evaluation of PHB production from activated sludge. Clean Technol Environ Policy.

[CR29] Obruca S, Benesova P, Marsalek L, Marova I (2015). Use of lignocellulosic materials for PHA production. Chem Biochem Eng Quaterly.

[CR30] Olaya-Abril A, Luque-Almagro VM, Manso I, Gates AJ, Moreno-Vivián C, Richardson DJ, Roldán MD (2017). Poly(3-hydroxybutyrate) hyperproduction by a global nitrogen regulator NtrB mutant strain of *Paracoccus denitrificans* PD1222. FEMS Microbiol Lett.

[CR31] Petrides D (2015) Bioprocess design and economics. In: Harrison RG, Todd PW, Rudge SR, Petrides D eds. Bioseparations Science and Engineering, 2nd edn. Oxford University Press, Chapter 11, ISBN 978-0-19-539181-7

[CR32] Qin Y, Wang H, Li X, Cheng JJ, Wu W (2017). Improving methane yield from organic fraction of municipal solid waste (OFMSW) with magnetic rice-straw biochar. Bioresour Technol.

[CR33] Ramos FD, Delpino CA, Villar MA, Diaz MS (2019). Design and optimization of poly(hydroxyalkanoate)s production plants using alternative substrates. Bioresour Technol.

[CR34] Raposo RS, de Almeida MC, de Oliveira MD, da Fonseca MM, Cesário MT (2017). A *Burkholderia sacchari* cell factory: production of poly-3-hydroxybutyrate, xylitol and xylonic acid from xylose-rich sugar mixtures. New Biotechnol..

[CR35] Rodriguez-Perez S, Serrano A, Pantión AA, Alonso-Fariñas B (2018). Challenges of scaling-up PHA production from waste streams. A review. J Environ Manage.

[CR36] Rosengart A, Cesário MT, de Almeida MCMD, Raposo RS, Espert A, de Apodaca ED, da Fonseca MMR (2015). Efficient P(3HB) extraction from *Burkholderia sacchari* cells using non-chlorinated solvents. Biochem Eng J.

[CR37] Rujnić-Sokele M, Pilipović A (2017). Challenges and opportunities of biodegradable plastics: a mini review. Waste Manag Res.

[CR38] Shahzad K, Kettl KH, Titz M, Koller M, Schnitzer H, Narodoslawsky M (2013). Comparison of ecological footprint for biobased PHA production from animal residues utilizing different energy resources. Clean Technol Environ Policy.

[CR44] Stegman P, Londo M, Junginger M (2020) The circular bioeconomy: Its elements and role in European bioeconomy clusters. Resources Conservation Recycling X 6, 100029

[CR40] Stavroula K, Simos M, Katherine-Joanne H (2020). Polyhydroxyalkanoates (PHAs) from household food waste: Research over the last decade. Int J Biotechnol Bioeng.

[CR41] Van Dael M, Kuppens T, Lizin S, Van Passel S, Fang Z, Smith RL, Qi X (2015). Techno-economic assessment methodology for ultrasonic production of biofuels. Production of biofuels and chemicals with ultrasound.

[CR42] Vandi L, Chan CM, Werker A, Richardson D, Laycock B, Pratt S (2018). Wood–PHA composites: mapping opportunities. Polymers.

[CR43] Xie CH, Yokota A (2005). Reclassification of *Alcaligenes latus* strains IAM 12599T and IAM 12664 and *Pseudomonas saccharophila* as *Azohydromonas lata* gen. nov. comb. nov., *Azohydromonas australica* sp. nov. and *Pelomonas saccharophila* gen. nov., comb. nov., respectively. Int J Syst Evol Microbiol.

