# Supplementary figures and images for: Economic and environmental assessment of bacterial poly(3-hydroxybutyrate) production from the organic fraction of municipal solid waste
Source: Bioresour Bioprocess. 2021 May 19;8(1):39. doi: 10.1186/s40643-021-00392-4 (PMC10992733; doi:10.1186/s40643-021-00392-4)

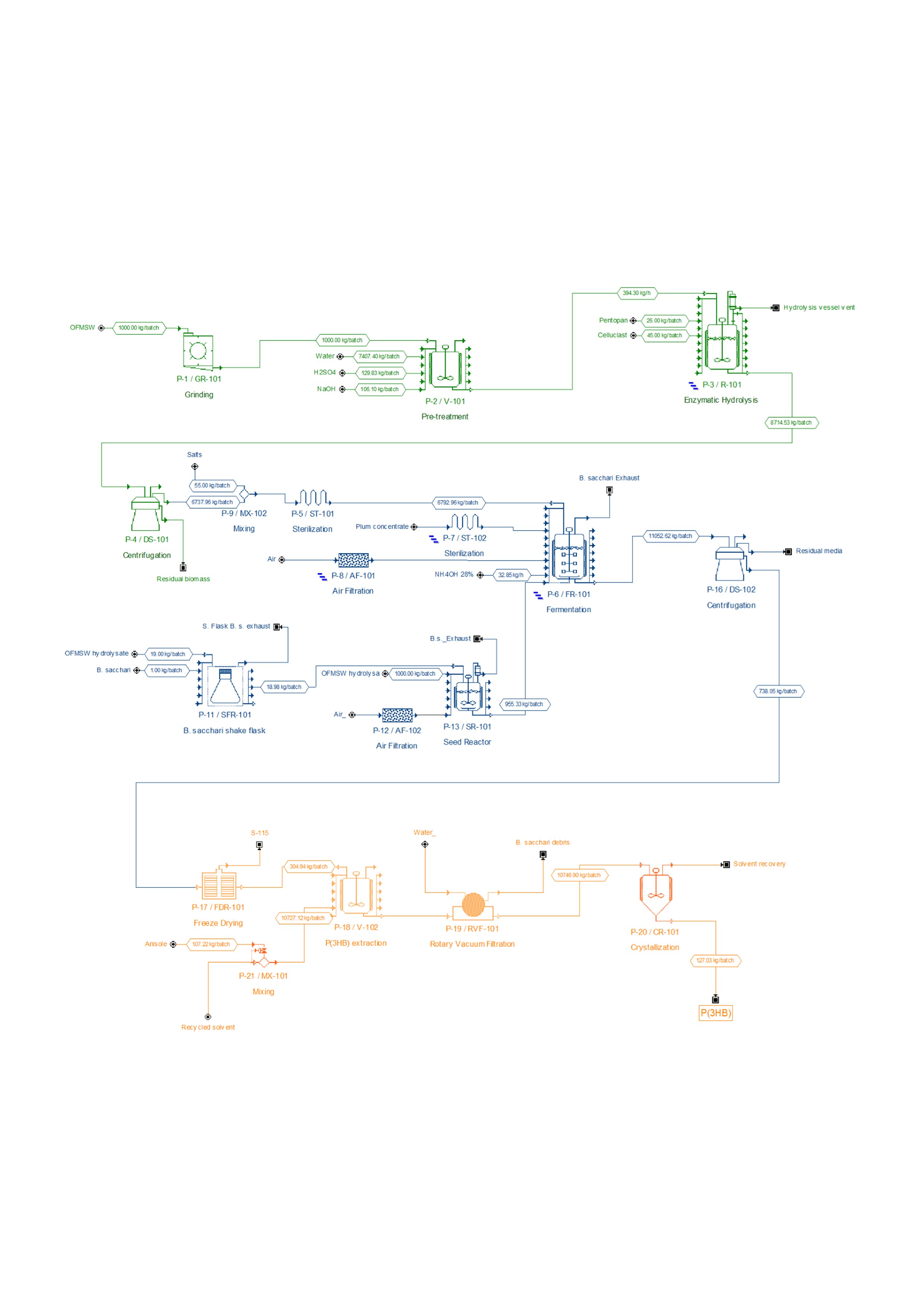

Supplement: Supplementary file 1 — Additional Figure 1. Flowsheet of the bacterial P3HB production process in Scenario 1. Green: thermo-chemical pre-treatment and enzymatic hydrolysis. Blue: bacterial process. Orange: extraction-separation. [file 40643_2021_392_MOESM1_ESM.jpg]

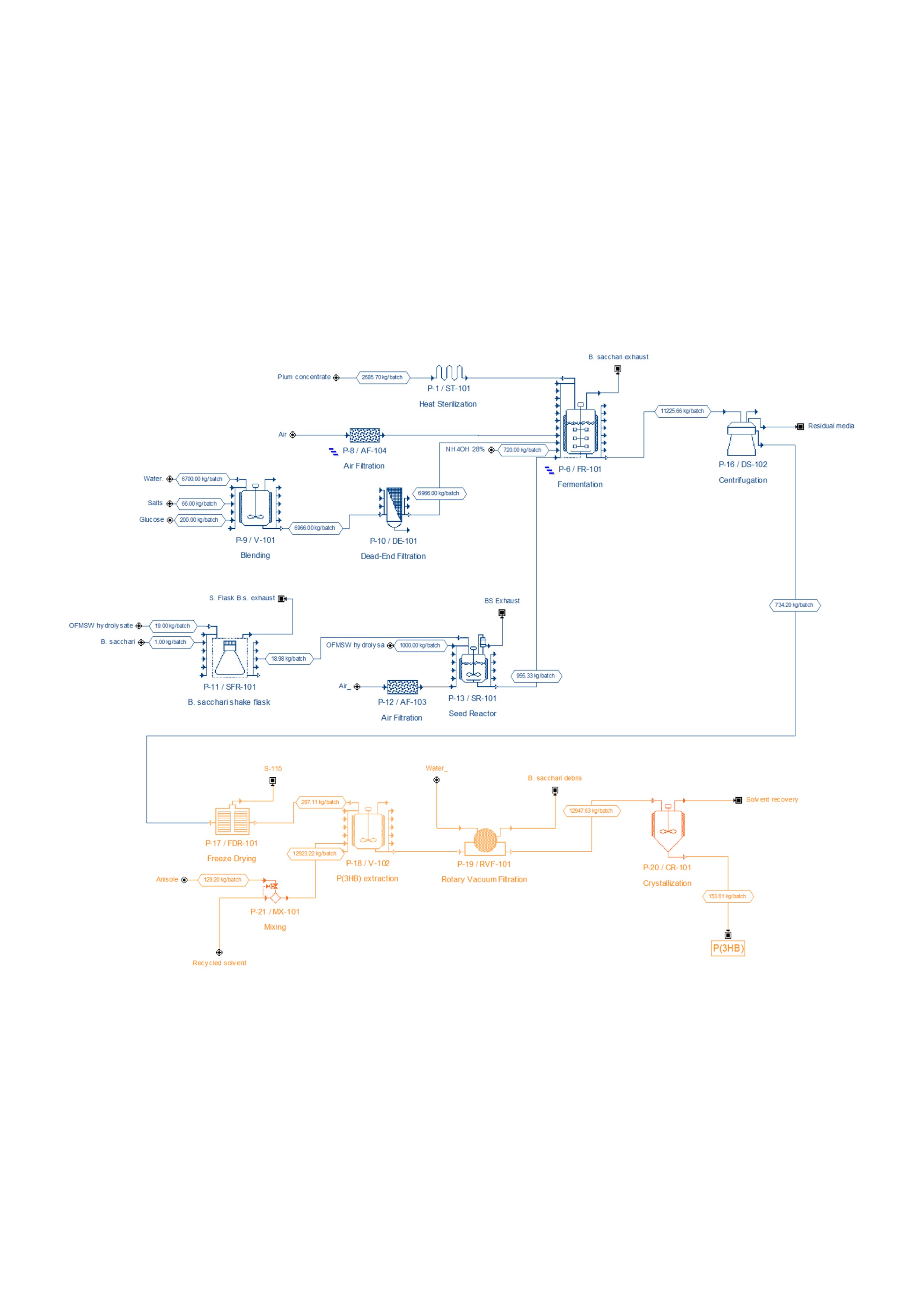

Supplement: Supplementary file 2 — Additional Figure 2. Flowsheet of the bacterial P3HB production process in Scenario 2. Green: thermo-chemical pre-treatment and enzymatic hydrolysis. Blue: bacterial process. Orange: extraction-separation. [file 40643_2021_392_MOESM2_ESM.jpg]
